# Tris(2-chloro­benz­yl)(1*H*-1,2,4-triazole-5-thiol­ato-κ*S*)tin(IV)–tris­(2-chloro­benz­yl)(4*H*-1,2,4-triazole-3-thiol­ato-κ*S*)tin(IV) (1/1)

**DOI:** 10.1107/S160053681003076X

**Published:** 2010-08-11

**Authors:** Thy Chun Keng, Kong Mun Lo, Seik Weng Ng

**Affiliations:** a Department of Chemistry, University of Malaya, 50603 Kuala Lumpur, Malaysia

## Abstract

Tris(2-chloro­benz­yl)tin hydroxide condenses with 3-mercapto-1,2,4-triazole to form the 1:1 cocrystal of the title compound, [Sn(C_7_H_6_Cl)_3_(C_2_H_2_N_3_S)]. The asymmetric unit contains two mol­ecules which differ only in the position of the nitro­gen-bound H atom of the triazole ring; one mol­ecule is linked to the other mol­ecule by an N—H⋯N hydrogen bond. In the second mol­ecule, two of the chloro­benzyl units are disordered over two positions in a 0.73 (1):0.27 (1) ratio. The Sn atom in both mol­ecules shows a distorted tetra­hedral SnSC_3_ coordination.

## Related literature

For comparison crystal structures, see: Aziz-ur-Rehman *et al.* (2006 ▶); Ma *et al.* (2007 ▶).
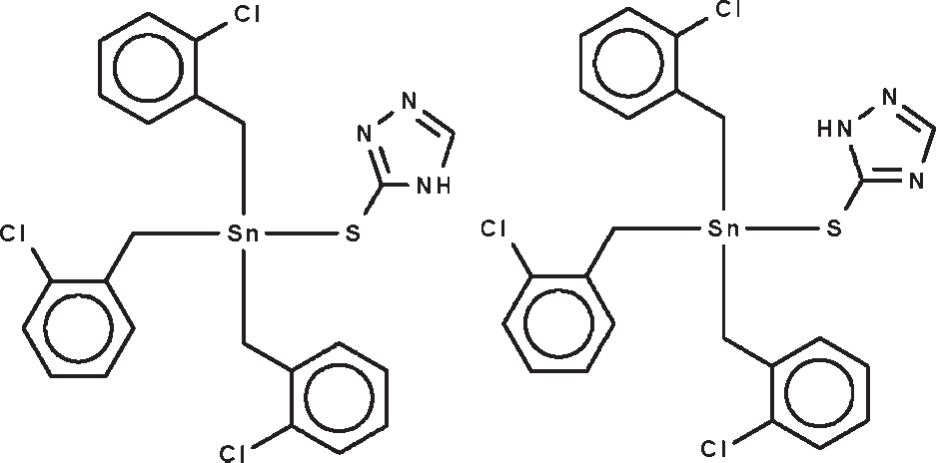


## Experimental

### 

#### Crystal data

[Sn(C_7_H_6_Cl)_3_(C_2_H_2_N_3_S)]
*M* *_r_* = 595.52
Triclinic, 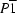

*a* = 9.696 (6) Å
*b* = 11.385 (5) Å
*c* = 23.670 (11) Å
α = 83.06 (2)°
β = 79.200 (18)°
γ = 68.559 (18)°
*V* = 2385 (2) Å^3^
*Z* = 4
Mo *K*α radiation
μ = 1.51 mm^−1^
*T* = 100 K
0.35 × 0.25 × 0.15 mm

#### Data collection

Bruker SMART APEX diffractometer
Absorption correction: multi-scan (*SADABS*; Sheldrick, 1996 ▶) *T*_min_ = 0.620, *T*_max_ = 0.805
22480 measured reflections
10780 independent reflections
9232 reflections with *I* > 2σ(*I*)
*R*_int_ = 0.031

#### Refinement

*R*[*F* ^2^ > 2σ(*F* ^2^)] = 0.034
*wR*(*F* ^2^) = 0.091
*S* = 1.01
10780 reflections
560 parameters
104 restraints
H-atom parameters constrained
Δρ_max_ = 1.41 e Å^−3^
Δρ_min_ = −0.81 e Å^−3^

### 

Data collection: *APEX2* (Bruker, 2009 ▶); cell refinement: *SAINT* (Bruker, 2009 ▶); data reduction: *SAINT*; program(s) used to solve structure: *SHELXS97* (Sheldrick, 2008 ▶); program(s) used to refine structure: *SHELXL97* (Sheldrick, 2008 ▶); molecular graphics: *X-SEED* (Barbour, 2001 ▶); software used to prepare material for publication: *publCIF* (Westrip, 2010 ▶).

## Supplementary Material

Crystal structure: contains datablocks global, I. DOI: 10.1107/S160053681003076X/xu5006sup1.cif

Structure factors: contains datablocks I. DOI: 10.1107/S160053681003076X/xu5006Isup2.hkl

Additional supplementary materials:  crystallographic information; 3D view; checkCIF report

## Figures and Tables

**Table 1:** Hydrogen-bond geometry (Å, °)

*D*—H⋯*A*	*D*—H	H⋯*A*	*D*⋯*A*	*D*—H⋯*A*
N2—H2⋯N5	0.86	2.07	2.916 (4)	170

**Fig. 1.**
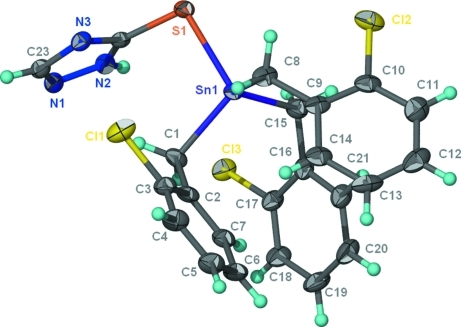
Thermal ellipsoid plot (Barbour, 2001) tris(2-chlorobenzyl)(1,2,4-triazoleyl-5-thiolato)tin at the 70% probability level; hydrogen atoms are drawn as spheres of arbitrary radius.

**Fig. 2.**
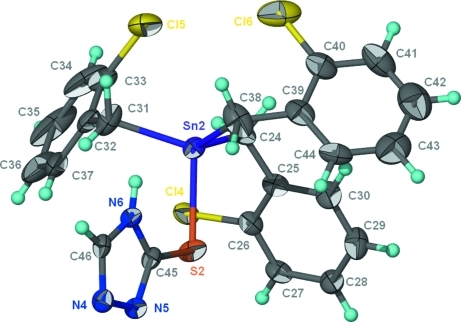
Thermal ellipsoid plot (Barbour, 2001) tris(2-chlorobenzyl)(1,3,4-triazole-2-thiolato)tin at the 70% probability level; hydrogen atoms are drawn as spheres of arbitrary radius.

## supplementary crystallographic information

### Comment

Triorganotin(IV) derivatives of thiols generally exists as tetrahedral molecules because the sulfidelinkage lowers the Lewis acidity of tin; for 1,2,4-triazolyl-3-thiolates, however, the nitrogen sites sometimes engage in intermolecular coordination and polymeric compounds are generated. Among the triorganotin 1,2,4-triazolyl-3-thiolates, only carbon- and nitrogen-substituted derivatives have been characterized by crystal structure analysis, *e.g.*, polymeric 4-methyl-1,2,4-triazole-3-thiolato)trimethyltin (Aziz-ur-Rehman *et al.*, 2006) and monomeric (4-methyl-5-trifluoromethyl-1,2,4-triazole-3-thiolato)triphenyltin (Ma *et al.*, 2007). 3-Mercapto-1,2,4-triazole itself has a nitrogen-bound hydrogen atom. It its condensation with tris(2-chlorobenzyl)tin hydroxide, the resulting product retains this hydrogen atom in the heterocyclic portion. The product is, in fact, a 1:1 co-crystal oftris(2-chlorobenzyl)(1,2,4-triazole-5-thiolato)tin(IV)–tris(2-\ chlorobenzyl)(1,3,4-triazole-2-thiolato)tin (Scheme I). The two molecular components are isoelectronic but differ only in the position of the nitrogen-bound hydrogen atom of the triazolyl ring. The tin atom in the two molecules show tetrahedral coordination. The first component (Fig. 1) is linked to the second component (Fig. 2) by an N–H···N hydrogen bond.

### Experimental

Tris(2-chlorobenzyl)tin hydroxide (0.5 g, 1 mmol) and 3-mercapto-1,2,4-triazole (0.1 g, 1 mmol) were dissolved in 50 m of ethanol. The solution was heated for 2 h. Colorless crystals separated from the filtered solution after several days.

### Refinement

Carbon-bound H-atoms were placed in calculated positions (C–H 0.95 to 0.99 Å) and were included in the refinement in the riding model approximation, with *U*(H) set to 1.2*U*(C).

For the heterocyclic ring in the first independent molecule, the amino H-atom was placed on the N2 atom as both the N1 and N3 atoms were within 2.8 Å of their symmetry-generated equivalents. The N2 atom forms a hydrogen bond to the N5 atom of the other independent molecule. For the heterocylic ring in the second independent molecule, a hydrogen atom placed on the N4 atom would be too close to the H23 atom of another molecule. Since the N5 atom is already an acceptor site, the N6 atom would then be the protonated atom. The N–H distances were set to 0.86 Å; the temperature factors were tied to those of the parent atoms.

The positioning of the amino H-atoms gives rise to different names for the two molecular species.

For the second molecule, two chlorobenzyl substitutents are disordered; the occupancies of the two could not be separately refined, and were assumed to be identical. The aromatic ring were refined as rigid hexagons of 1.39 Å sides. The tin–carbon distances for each pair of Sn–C bonds were restrained to within 0.01 Å of each other. The carbon_methylene_–carbon_phenylene_ distances were restrained to 1.50±0.01 Å and the carbon–chlorine distances were restrained to 1.75±0.01 Å. The temperature factors of the primed atoms were set to those of the unprimed ones; the anisotropic temperature factors of the disordered carbon atoms were restrained to be nearly isotropic. The disorder refined to a 79:21 ratio.

The final difference Fourier map had a highest peak in the vicinity of C26.

### Crystal data

**Table:**

[Sn(C_7_H_6_Cl)_3_(C_2_H_2_N_3_S)]	*Z* = 4
*M**_r_* = 595.52	*F*(000) = 1184
Triclinic, *P*1	*D*_x_ = 1.658 Mg m^−^^3^
Hall symbol: -P 1	Mo *K*α radiation, λ = 0.71073 Å
*a* = 9.696 (6) Å	Cell parameters from 9913 reflections
*b* = 11.385 (5) Å	θ = 2.2–28.3°
*c* = 23.670 (11) Å	µ = 1.51 mm^−^^1^
α = 83.06 (2)°	*T* = 100 K
β = 79.200 (18)°	Polycrystals, colorless
γ = 68.559 (18)°	0.35 × 0.25 × 0.15 mm
*V* = 2385 (2) Å^3^	

### Data collection

**Table:**

Bruker SMART APEX diffractometer	10780 independent reflections
Radiation source: fine-focus sealed tube	9232 reflections with *I* > 2σ(*I*)
graphite	*R*_int_ = 0.031
ω scans	θ_max_ = 27.5°, θ_min_ = 0.9°
Absorption correction: multi-scan (*SADABS*; Sheldrick, 1996)	*h* = −12→12
*T*_min_ = 0.620, *T*_max_ = 0.805	*k* = −14→14
22480 measured reflections	*l* = −28→30

### Refinement

**Table:**

Refinement on *F*^2^	Primary atom site location: structure-invariant direct methods
Least-squares matrix: full	Secondary atom site location: difference Fourier map
*R*[*F*^2^ > 2σ(*F*^2^)] = 0.034	Hydrogen site location: inferred from neighbouring sites
*wR*(*F*^2^) = 0.091	H-atom parameters constrained
*S* = 1.01	*w* = 1/[σ^2^(*F*_o_^2^) + (0.0391*P*)^2^ + 3.8486*P*] where *P* = (*F*_o_^2^ + 2*F*_c_^2^)/3
10780 reflections	(Δ/σ)_max_ = 0.001
560 parameters	Δρ_max_ = 1.41 e Å^−^^3^
104 restraints	Δρ_min_ = −0.81 e Å^−^^3^

### Fractional atomic coordinates and isotropic or equivalent isotropic displacement parameters (Å^2^)

**Table:**

	*x*	*y*	*z*	*U*_iso_*/*U*_eq_	Occ. (<1)
Sn1	0.64284 (2)	0.717035 (18)	0.636789 (9)	0.01646 (6)	
Sn2	0.82300 (2)	0.96070 (2)	0.862329 (9)	0.02124 (6)	
Cl1	0.62336 (10)	0.70680 (8)	0.46944 (4)	0.03049 (18)	
Cl2	0.33404 (9)	0.65825 (8)	0.75698 (4)	0.03087 (18)	
Cl3	1.04011 (8)	0.67040 (7)	0.67373 (4)	0.02481 (16)	
Cl4	0.5643 (5)	0.7542 (3)	0.85926 (18)	0.0329 (5)	0.73 (1)
Cl4'	0.5454 (17)	0.7760 (9)	0.8523 (6)	0.0329 (5)	0.27
Cl5	0.89631 (14)	0.75253 (11)	0.99446 (4)	0.0507 (3)	
Cl6	0.7594 (2)	1.12134 (15)	1.00302 (6)	0.0495 (4)	0.73 (1)
Cl6'	0.8829 (6)	1.0551 (4)	0.98374 (18)	0.0495 (4)	0.27
S1	0.54143 (8)	0.95007 (7)	0.63370 (3)	0.02102 (15)	
S2	0.75356 (9)	0.97103 (9)	0.76652 (3)	0.02605 (17)	
N1	0.8550 (3)	0.9981 (2)	0.51761 (11)	0.0189 (5)	
N2	0.7975 (3)	0.9781 (2)	0.57403 (11)	0.0175 (5)	
H2	0.8399	0.9692	0.6039	0.021*	
N3	0.6304 (3)	0.9951 (2)	0.51800 (11)	0.0185 (5)	
N4	1.0966 (3)	0.9713 (3)	0.65034 (11)	0.0222 (5)	
N5	0.9523 (3)	0.9742 (2)	0.66902 (11)	0.0193 (5)	
N6	1.0439 (3)	0.9690 (2)	0.74744 (11)	0.0191 (5)	
H6	1.0547	0.9679	0.7828	0.023*	
C1	0.8193 (3)	0.6591 (3)	0.56364 (13)	0.0201 (6)	
H1A	0.8068	0.7297	0.5339	0.024*	
H1B	0.9180	0.6384	0.5760	0.024*	
C2	0.8145 (3)	0.5466 (3)	0.53875 (13)	0.0193 (6)	
C3	0.7299 (3)	0.5566 (3)	0.49549 (14)	0.0221 (6)	
C4	0.7265 (4)	0.4518 (3)	0.47200 (15)	0.0258 (7)	
H4	0.6698	0.4624	0.4419	0.031*	
C5	0.8065 (4)	0.3317 (3)	0.49289 (16)	0.0285 (7)	
H5	0.8041	0.2593	0.4775	0.034*	
C6	0.8897 (4)	0.3182 (3)	0.53623 (15)	0.0268 (7)	
H6A	0.9438	0.2360	0.5510	0.032*	
C7	0.8948 (3)	0.4238 (3)	0.55845 (14)	0.0227 (6)	
H7	0.9542	0.4124	0.5877	0.027*	
C8	0.4607 (3)	0.6561 (3)	0.62835 (15)	0.0225 (6)	
H8A	0.3640	0.7207	0.6434	0.027*	
H8B	0.4611	0.6488	0.5871	0.027*	
C9	0.4733 (3)	0.5316 (3)	0.66031 (14)	0.0212 (6)	
C10	0.4184 (3)	0.5209 (3)	0.71877 (15)	0.0236 (7)	
C11	0.4267 (4)	0.4066 (3)	0.74811 (16)	0.0287 (7)	
H11	0.3848	0.4037	0.7877	0.034*	
C12	0.4971 (4)	0.2957 (3)	0.71903 (17)	0.0317 (8)	
H12	0.5040	0.2163	0.7386	0.038*	
C13	0.5569 (4)	0.3018 (3)	0.66141 (17)	0.0300 (8)	
H13	0.6071	0.2263	0.6415	0.036*	
C14	0.5437 (3)	0.4178 (3)	0.63259 (15)	0.0246 (7)	
H14	0.5836	0.4204	0.5928	0.029*	
C15	0.7312 (3)	0.6453 (3)	0.71648 (14)	0.0225 (6)	
H15A	0.7609	0.7092	0.7309	0.027*	
H15B	0.6532	0.6274	0.7460	0.027*	
C16	0.8643 (3)	0.5269 (3)	0.70591 (13)	0.0212 (6)	
C17	1.0099 (3)	0.5266 (3)	0.68595 (14)	0.0213 (6)	
C18	1.1331 (4)	0.4161 (3)	0.67527 (15)	0.0254 (7)	
H18	1.2305	0.4194	0.6622	0.030*	
C19	1.1113 (4)	0.3010 (3)	0.68398 (16)	0.0307 (8)	
H19	1.1941	0.2246	0.6770	0.037*	
C20	0.9681 (4)	0.2977 (3)	0.70295 (16)	0.0292 (7)	
H20	0.9529	0.2189	0.7088	0.035*	
C21	0.8477 (4)	0.4085 (3)	0.71337 (14)	0.0247 (7)	
H21	0.7505	0.4044	0.7260	0.030*	
C22	0.6639 (3)	0.9754 (3)	0.57264 (13)	0.0160 (5)	
C23	0.7537 (3)	1.0078 (3)	0.48527 (13)	0.0196 (6)	
H23	0.7663	1.0218	0.4445	0.024*	
C24	0.6163 (7)	0.955 (2)	0.9154 (3)	0.023 (2)	0.73 (1)
H24A	0.6316	0.8675	0.9309	0.028*	0.73 (1)
H24B	0.5933	1.0084	0.9486	0.028*	0.73 (1)
C25	0.4840 (10)	1.0009 (6)	0.8834 (5)	0.0233 (10)	0.73 (1)
C26	0.4562 (9)	0.9136 (4)	0.8551 (5)	0.0232 (9)	0.73 (1)
C27	0.3408 (7)	0.9537 (3)	0.8223 (3)	0.0251 (10)	0.73 (1)
H27	0.3218	0.8940	0.8029	0.030*	0.73 (1)
C28	0.2531 (5)	1.0810 (4)	0.8177 (2)	0.0253 (11)	0.73 (1)
H28	0.1741	1.1084	0.7953	0.030*	0.73 (1)
C29	0.2808 (6)	1.1683 (3)	0.8460 (3)	0.0342 (13)	0.73 (1)
H29	0.2209	1.2553	0.8429	0.041*	0.73 (1)
C30	0.3963 (9)	1.1282 (5)	0.8788 (4)	0.0297 (11)	0.73 (1)
H30	0.4153	1.1879	0.8982	0.036*	0.73 (1)
C24'	0.6154 (19)	0.969 (6)	0.9188 (9)	0.023 (2)	0.27
H24C	0.6250	0.8847	0.9375	0.028*	0.27 (1)
H24D	0.5946	1.0286	0.9493	0.028*	0.27 (1)
C25'	0.489 (3)	1.0132 (19)	0.8843 (15)	0.0233 (10)	0.27
C26'	0.448 (3)	0.9380 (11)	0.8533 (14)	0.0232 (9)	0.27
C27'	0.325 (2)	0.9913 (12)	0.8246 (10)	0.0251 (10)	0.27
H27'	0.2972	0.9400	0.8034	0.030*	0.27 (1)
C28'	0.2423 (17)	1.1199 (13)	0.8269 (7)	0.0253 (11)	0.27
H28'	0.1580	1.1563	0.8073	0.030*	0.27 (1)
C29'	0.283 (2)	1.1950 (10)	0.8579 (8)	0.0342 (13)	0.27
H29'	0.2264	1.2829	0.8595	0.041*	0.27 (1)
C30'	0.406 (3)	1.1417 (18)	0.8866 (12)	0.0297 (11)	0.27
H30'	0.4339	1.1931	0.9077	0.036*	0.27 (1)
C31	1.0163 (4)	0.7921 (4)	0.86879 (17)	0.0355 (8)	
H31A	1.0840	0.7805	0.8314	0.043*	
H31B	1.0716	0.8012	0.8982	0.043*	
C32	0.9715 (4)	0.6791 (3)	0.88492 (15)	0.0281 (7)	
C33	0.9136 (4)	0.6515 (4)	0.94113 (16)	0.0309 (8)	
C34	0.8664 (5)	0.5499 (5)	0.9566 (2)	0.0546 (14)	
H34	0.8277	0.5334	0.9954	0.066*	
C35	0.8772 (7)	0.4727 (5)	0.9137 (3)	0.075 (2)	
H35	0.8465	0.4015	0.9231	0.090*	
C36	0.9321 (7)	0.4986 (4)	0.8574 (3)	0.0683 (18)	
H36	0.9370	0.4462	0.8282	0.082*	
C37	0.9799 (5)	0.5996 (4)	0.84321 (19)	0.0441 (10)	
H37	1.0193	0.6153	0.8043	0.053*	
C38	0.8610 (11)	1.1337 (7)	0.8715 (5)	0.038 (2)	0.73 (1)
H38A	0.9382	1.1147	0.8967	0.046*	0.73 (1)
H38B	0.9007	1.1640	0.8332	0.046*	0.73 (1)
C39	0.7219 (3)	1.2377 (3)	0.89641 (13)	0.0305 (10)	0.73 (1)
C40	0.6696 (4)	1.2378 (3)	0.95525 (12)	0.0314 (11)	0.73 (1)
C41	0.5421 (4)	1.3355 (3)	0.97739 (11)	0.0455 (14)	0.73 (1)
H41	0.5064	1.3355	1.0176	0.055*	0.73 (1)
C42	0.4669 (4)	1.4330 (3)	0.94069 (16)	0.0513 (16)	0.73 (1)
H42	0.3798	1.4998	0.9558	0.062*	0.73 (1)
C43	0.5192 (4)	1.4329 (3)	0.88185 (15)	0.0451 (13)	0.73 (1)
H43	0.4678	1.4996	0.8568	0.054*	0.73 (1)
C44	0.6467 (4)	1.3353 (3)	0.85971 (10)	0.0289 (10)	0.73 (1)
H44	0.6824	1.3352	0.8195	0.035*	0.73 (1)
C38'	0.863 (3)	1.137 (2)	0.8598 (17)	0.038 (2)	0.27
H38C	0.9621	1.1216	0.8704	0.046*	0.27 (1)
H38D	0.8597	1.1789	0.8205	0.046*	0.27 (1)
C39'	0.7410 (11)	1.2202 (10)	0.9021 (4)	0.0305 (10)	0.27
C40'	0.7423 (10)	1.1869 (8)	0.9605 (4)	0.0314 (11)	0.27
C41'	0.6279 (13)	1.2578 (10)	1.0009 (3)	0.0455 (14)	0.27
H41'	0.6287	1.2350	1.0408	0.055*	0.27 (1)
C42'	0.5121 (11)	1.3620 (10)	0.9829 (4)	0.0513 (16)	0.27
H42'	0.4339	1.4104	1.0105	0.062*	0.27 (1)
C43'	0.5108 (11)	1.3953 (9)	0.9245 (5)	0.0451 (13)	0.27
H43'	0.4317	1.4665	0.9122	0.054*	0.27 (1)
C44'	0.6253 (13)	1.3244 (10)	0.8841 (3)	0.0289 (10)	0.27
H44'	0.6244	1.3472	0.8441	0.035*	0.27 (1)
C45	0.9244 (3)	0.9717 (3)	0.72669 (13)	0.0188 (6)	
C46	1.1446 (3)	0.9682 (3)	0.69898 (13)	0.0203 (6)	
H46	1.2430	0.9656	0.7002	0.024*	

### Atomic displacement parameters (Å^2^)

**Table:**

	*U*^11^	*U*^22^	*U*^33^	*U*^12^	*U*^13^	*U*^23^
Sn1	0.01163 (10)	0.01449 (10)	0.02346 (11)	−0.00542 (8)	−0.00056 (7)	−0.00253 (7)
Sn2	0.01800 (11)	0.02336 (12)	0.02437 (12)	−0.00923 (9)	−0.00485 (8)	−0.00019 (8)
Cl1	0.0314 (4)	0.0200 (4)	0.0403 (5)	−0.0047 (3)	−0.0153 (4)	−0.0016 (3)
Cl2	0.0206 (4)	0.0294 (4)	0.0421 (5)	−0.0091 (3)	0.0041 (3)	−0.0138 (4)
Cl3	0.0204 (4)	0.0200 (4)	0.0366 (4)	−0.0100 (3)	−0.0051 (3)	−0.0006 (3)
Cl4	0.0486 (15)	0.0211 (13)	0.0303 (13)	−0.0117 (11)	−0.0123 (8)	0.0018 (9)
Cl4'	0.0486 (15)	0.0211 (13)	0.0303 (13)	−0.0117 (11)	−0.0123 (8)	0.0018 (9)
Cl5	0.0627 (7)	0.0537 (7)	0.0326 (5)	−0.0101 (5)	−0.0222 (5)	−0.0013 (4)
Cl6	0.0773 (10)	0.0460 (8)	0.0423 (8)	−0.0346 (8)	−0.0306 (7)	0.0080 (6)
Cl6'	0.0773 (10)	0.0460 (8)	0.0423 (8)	−0.0346 (8)	−0.0306 (7)	0.0080 (6)
S1	0.0170 (3)	0.0154 (3)	0.0278 (4)	−0.0055 (3)	0.0039 (3)	−0.0023 (3)
S2	0.0184 (4)	0.0399 (5)	0.0237 (4)	−0.0159 (3)	−0.0053 (3)	0.0054 (3)
N1	0.0157 (12)	0.0193 (13)	0.0227 (13)	−0.0082 (10)	−0.0029 (10)	0.0010 (10)
N2	0.0150 (12)	0.0202 (13)	0.0197 (12)	−0.0079 (10)	−0.0057 (9)	0.0004 (10)
N3	0.0166 (12)	0.0160 (12)	0.0238 (13)	−0.0063 (10)	−0.0056 (10)	0.0010 (10)
N4	0.0186 (13)	0.0233 (14)	0.0240 (13)	−0.0076 (11)	−0.0018 (10)	−0.0005 (11)
N5	0.0171 (12)	0.0220 (13)	0.0209 (13)	−0.0094 (10)	−0.0034 (10)	−0.0001 (10)
N6	0.0180 (12)	0.0207 (13)	0.0194 (12)	−0.0074 (10)	−0.0048 (10)	0.0006 (10)
C1	0.0141 (13)	0.0236 (15)	0.0231 (15)	−0.0086 (12)	0.0025 (11)	−0.0054 (12)
C2	0.0118 (13)	0.0190 (15)	0.0262 (15)	−0.0056 (11)	0.0028 (11)	−0.0061 (12)
C3	0.0164 (14)	0.0185 (15)	0.0287 (16)	−0.0036 (12)	−0.0018 (12)	−0.0019 (12)
C4	0.0245 (16)	0.0232 (16)	0.0321 (18)	−0.0094 (14)	−0.0043 (13)	−0.0077 (13)
C5	0.0268 (17)	0.0204 (16)	0.0389 (19)	−0.0113 (14)	0.0041 (14)	−0.0091 (14)
C6	0.0177 (15)	0.0185 (15)	0.0381 (19)	−0.0026 (12)	0.0038 (13)	−0.0030 (13)
C7	0.0149 (14)	0.0228 (16)	0.0273 (16)	−0.0048 (12)	0.0023 (12)	−0.0040 (13)
C8	0.0128 (14)	0.0195 (15)	0.0379 (18)	−0.0084 (12)	−0.0041 (12)	−0.0025 (13)
C9	0.0109 (13)	0.0173 (14)	0.0369 (18)	−0.0060 (11)	−0.0030 (12)	−0.0050 (13)
C10	0.0122 (13)	0.0195 (15)	0.0388 (18)	−0.0051 (12)	−0.0004 (12)	−0.0076 (13)
C11	0.0223 (16)	0.0274 (17)	0.0395 (19)	−0.0135 (14)	−0.0051 (14)	0.0022 (15)
C12	0.0230 (17)	0.0204 (16)	0.054 (2)	−0.0091 (14)	−0.0120 (15)	0.0055 (15)
C13	0.0160 (15)	0.0170 (15)	0.057 (2)	−0.0032 (13)	−0.0052 (15)	−0.0094 (15)
C14	0.0130 (14)	0.0226 (16)	0.0385 (18)	−0.0064 (12)	−0.0019 (12)	−0.0057 (14)
C15	0.0186 (15)	0.0229 (16)	0.0248 (16)	−0.0067 (13)	−0.0005 (12)	−0.0032 (12)
C16	0.0201 (15)	0.0225 (16)	0.0221 (15)	−0.0089 (13)	−0.0053 (12)	0.0023 (12)
C17	0.0192 (15)	0.0175 (15)	0.0281 (16)	−0.0069 (12)	−0.0058 (12)	0.0002 (12)
C18	0.0148 (14)	0.0231 (16)	0.0362 (18)	−0.0040 (13)	−0.0070 (13)	0.0020 (13)
C19	0.0225 (17)	0.0190 (16)	0.044 (2)	0.0007 (13)	−0.0085 (14)	0.0031 (14)
C20	0.0308 (18)	0.0195 (16)	0.0377 (19)	−0.0102 (14)	−0.0086 (15)	0.0068 (14)
C21	0.0203 (15)	0.0245 (16)	0.0304 (17)	−0.0104 (13)	−0.0053 (13)	0.0048 (13)
C22	0.0122 (13)	0.0126 (13)	0.0227 (14)	−0.0038 (11)	−0.0020 (11)	−0.0017 (11)
C23	0.0179 (14)	0.0171 (14)	0.0226 (15)	−0.0051 (12)	−0.0035 (11)	0.0011 (11)
C24	0.0267 (16)	0.028 (5)	0.0201 (18)	−0.0167 (16)	−0.0017 (13)	−0.005 (2)
C25	0.0197 (16)	0.034 (2)	0.0220 (16)	−0.0179 (17)	0.0001 (12)	−0.0017 (18)
C26	0.0288 (19)	0.021 (2)	0.0219 (17)	−0.014 (2)	−0.0002 (14)	0.001 (3)
C27	0.029 (2)	0.028 (3)	0.0266 (19)	−0.019 (3)	−0.0029 (17)	−0.007 (3)
C28	0.0201 (18)	0.028 (3)	0.031 (3)	−0.010 (2)	−0.0047 (17)	−0.010 (2)
C29	0.0265 (19)	0.028 (3)	0.051 (4)	−0.011 (2)	−0.005 (2)	−0.012 (2)
C30	0.019 (2)	0.037 (2)	0.039 (3)	−0.0146 (18)	−0.0045 (17)	−0.008 (2)
C24'	0.0267 (16)	0.028 (5)	0.0201 (18)	−0.0167 (16)	−0.0017 (13)	−0.005 (2)
C25'	0.0197 (16)	0.034 (2)	0.0220 (16)	−0.0179 (17)	0.0001 (12)	−0.0017 (18)
C26'	0.0288 (19)	0.021 (2)	0.0219 (17)	−0.014 (2)	−0.0002 (14)	0.001 (3)
C27'	0.029 (2)	0.028 (3)	0.0266 (19)	−0.019 (3)	−0.0029 (17)	−0.007 (3)
C28'	0.0201 (18)	0.028 (3)	0.031 (3)	−0.010 (2)	−0.0047 (17)	−0.010 (2)
C29'	0.0265 (19)	0.028 (3)	0.051 (4)	−0.011 (2)	−0.005 (2)	−0.012 (2)
C30'	0.019 (2)	0.037 (2)	0.039 (3)	−0.0146 (18)	−0.0045 (17)	−0.008 (2)
C31	0.0222 (17)	0.034 (2)	0.043 (2)	−0.0055 (15)	−0.0066 (15)	0.0122 (16)
C32	0.0248 (17)	0.0248 (17)	0.0314 (18)	−0.0021 (14)	−0.0132 (14)	0.0036 (14)
C33	0.0321 (19)	0.0328 (19)	0.0324 (18)	−0.0138 (16)	−0.0168 (15)	0.0074 (15)
C34	0.052 (3)	0.062 (3)	0.066 (3)	−0.039 (2)	−0.038 (2)	0.039 (2)
C35	0.085 (4)	0.037 (3)	0.135 (6)	−0.038 (3)	−0.087 (4)	0.033 (3)
C36	0.087 (4)	0.031 (2)	0.099 (4)	−0.005 (2)	−0.074 (4)	−0.005 (3)
C37	0.044 (2)	0.034 (2)	0.044 (2)	0.0098 (18)	−0.0240 (19)	−0.0100 (17)
C38	0.038 (2)	0.039 (2)	0.045 (5)	−0.0258 (19)	0.009 (3)	−0.015 (2)
C39	0.034 (2)	0.034 (2)	0.033 (2)	−0.0213 (19)	−0.0053 (17)	−0.0086 (17)
C40	0.039 (3)	0.030 (3)	0.033 (2)	−0.019 (2)	−0.006 (2)	−0.009 (2)
C41	0.058 (4)	0.040 (3)	0.043 (3)	−0.026 (3)	0.008 (2)	−0.017 (2)
C42	0.054 (4)	0.046 (3)	0.058 (4)	−0.021 (3)	−0.001 (3)	−0.021 (3)
C43	0.052 (3)	0.039 (3)	0.057 (3)	−0.023 (3)	−0.024 (3)	−0.001 (2)
C44	0.048 (3)	0.036 (2)	0.013 (2)	−0.028 (2)	−0.006 (2)	0.003 (2)
C38'	0.038 (2)	0.039 (2)	0.045 (5)	−0.0258 (19)	0.009 (3)	−0.015 (2)
C39'	0.034 (2)	0.034 (2)	0.033 (2)	−0.0213 (19)	−0.0053 (17)	−0.0086 (17)
C40'	0.039 (3)	0.030 (3)	0.033 (2)	−0.019 (2)	−0.006 (2)	−0.009 (2)
C41'	0.058 (4)	0.040 (3)	0.043 (3)	−0.026 (3)	0.008 (2)	−0.017 (2)
C42'	0.054 (4)	0.046 (3)	0.058 (4)	−0.021 (3)	−0.001 (3)	−0.021 (3)
C43'	0.052 (3)	0.039 (3)	0.057 (3)	−0.023 (3)	−0.024 (3)	−0.001 (2)
C44'	0.048 (3)	0.036 (2)	0.013 (2)	−0.028 (2)	−0.006 (2)	0.003 (2)
C45	0.0167 (14)	0.0158 (14)	0.0241 (15)	−0.0062 (11)	−0.0045 (11)	0.0020 (11)
C46	0.0145 (14)	0.0185 (15)	0.0254 (16)	−0.0037 (12)	−0.0019 (11)	−0.0012 (12)

### Geometric parameters (Å, °)

**Table:**

Sn1—C15	2.163 (3)	C20—H20	0.9500
Sn1—C1	2.168 (3)	C21—H21	0.9500
Sn1—C8	2.168 (3)	C23—H23	0.9500
Sn1—S1	2.4667 (14)	C24—C25	1.508 (5)
Sn2—C31	2.151 (4)	C24—H24A	0.9900
Sn2—C38	2.174 (5)	C24—H24B	0.9900
Sn2—C38'	2.174 (8)	C25—C26	1.3900
Sn2—C24'	2.174 (8)	C25—C30	1.3900
Sn2—C24	2.174 (4)	C26—C27	1.3900
Sn2—S2	2.4617 (13)	C27—C28	1.3900
Cl1—C3	1.751 (3)	C27—H27	0.9500
Cl2—C10	1.749 (3)	C28—C29	1.3900
Cl3—C17	1.748 (3)	C28—H28	0.9500
Cl4—C26	1.734 (3)	C29—C30	1.3900
Cl4'—C26'	1.738 (9)	C29—H29	0.9500
Cl5—C33	1.754 (4)	C30—H30	0.9500
Cl6—C40	1.720 (3)	C24'—C25'	1.501 (10)
Cl6'—C40'	1.732 (7)	C24'—H24C	0.9900
Cl6'—Cl6'^i^	2.379 (9)	C24'—H24D	0.9900
S1—C22	1.757 (3)	C25'—C26'	1.3900
S2—C45	1.748 (3)	C25'—C30'	1.3900
N1—C23	1.321 (4)	C26'—C27'	1.3900
N1—N2	1.374 (3)	C27'—C28'	1.3900
N2—C22	1.314 (4)	C27'—H27'	0.9500
N2—H2	0.8600	C28'—C29'	1.3900
N3—C23	1.343 (4)	C28'—H28'	0.9500
N3—C22	1.365 (4)	C29'—C30'	1.3900
N4—C46	1.312 (4)	C29'—H29'	0.9500
N4—N5	1.376 (4)	C30'—H30'	0.9500
N5—C45	1.340 (4)	C31—C32	1.489 (5)
N6—C45	1.329 (4)	C31—H31A	0.9900
N6—C46	1.359 (4)	C31—H31B	0.9900
N6—H6	0.8600	C32—C37	1.391 (5)
C1—C2	1.493 (4)	C32—C33	1.390 (5)
C1—H1A	0.9900	C33—C34	1.379 (5)
C1—H1B	0.9900	C34—C35	1.387 (8)
C2—C3	1.397 (4)	C34—H34	0.9500
C2—C7	1.400 (4)	C35—C36	1.378 (9)
C3—C4	1.389 (4)	C35—H35	0.9500
C4—C5	1.386 (5)	C36—C37	1.374 (7)
C4—H4	0.9500	C36—H36	0.9500
C5—C6	1.381 (5)	C37—H37	0.9500
C5—H5	0.9500	C38—C39	1.507 (7)
C6—C7	1.389 (5)	C38—H38A	0.9900
C6—H6A	0.9500	C38—H38B	0.9900
C7—H7	0.9500	C39—C40	1.3900
C8—C9	1.499 (4)	C39—C44	1.3900
C8—H8A	0.9900	C40—C41	1.3900
C8—H8B	0.9900	C41—C42	1.3900
C9—C10	1.394 (5)	C41—H41	0.9500
C9—C14	1.402 (4)	C42—C43	1.3900
C10—C11	1.382 (5)	C42—H42	0.9500
C11—C12	1.392 (5)	C43—C44	1.3900
C11—H11	0.9500	C43—H43	0.9500
C12—C13	1.383 (5)	C44—H44	0.9500
C12—H12	0.9500	C38'—C39'	1.501 (10)
C13—C14	1.385 (5)	C38'—H38C	0.9900
C13—H13	0.9500	C38'—H38D	0.9900
C14—H14	0.9500	C39'—C40'	1.3900
C15—C16	1.495 (4)	C39'—C44'	1.3900
C15—H15A	0.9900	C40'—C41'	1.3900
C15—H15B	0.9900	C41'—C42'	1.3900
C16—C21	1.401 (4)	C41'—H41'	0.9500
C16—C17	1.401 (4)	C42'—C43'	1.3900
C17—C18	1.393 (5)	C42'—H42'	0.9500
C18—C19	1.388 (5)	C43'—C44'	1.3900
C18—H18	0.9500	C43'—H43'	0.9500
C19—C20	1.390 (5)	C44'—H44'	0.9500
C19—H19	0.9500	C46—H46	0.9500
C20—C21	1.381 (5)		
			
C15—Sn1—C1	110.37 (12)	C26—C25—C24	118.5 (9)
C15—Sn1—C8	112.66 (12)	C30—C25—C24	121.3 (9)
C1—Sn1—C8	109.34 (13)	C27—C26—C25	120.0
C15—Sn1—S1	109.39 (9)	C27—C26—Cl4	118.7 (4)
C1—Sn1—S1	108.10 (9)	C25—C26—Cl4	121.3 (4)
C8—Sn1—S1	106.83 (9)	C26—C27—C28	120.0
C31—Sn2—C38	113.9 (3)	C26—C27—H27	120.0
C31—Sn2—C38'	115.4 (10)	C28—C27—H27	120.0
C38—Sn2—C38'	7.2 (14)	C27—C28—C29	120.0
C31—Sn2—C24'	117.7 (15)	C27—C28—H28	120.0
C38—Sn2—C24'	106.8 (19)	C29—C28—H28	120.0
C38'—Sn2—C24'	110.8 (19)	C30—C29—C28	120.0
C31—Sn2—C24	115.2 (5)	C30—C29—H29	120.0
C38—Sn2—C24	111.5 (6)	C28—C29—H29	120.0
C38'—Sn2—C24	115.5 (7)	C29—C30—C25	120.0
C24'—Sn2—C24	5(2)	C29—C30—H30	120.0
C31—Sn2—S2	107.24 (12)	C25—C30—H30	120.0
C38—Sn2—S2	108.5 (4)	C25'—C24'—Sn2	109.5 (18)
C38'—Sn2—S2	101.5 (13)	C25'—C24'—H24C	109.8
C24'—Sn2—S2	101.8 (7)	Sn2—C24'—H24C	109.8
C24—Sn2—S2	99.2 (2)	C25'—C24'—H24D	109.8
C40'—Cl6'—Cl6'^i^	155.1 (5)	Sn2—C24'—H24D	109.8
C22—S1—Sn1	97.09 (10)	H24C—C24'—H24D	108.2
C45—S2—Sn2	96.57 (11)	C26'—C25'—C30'	120.0
C23—N1—N2	108.2 (2)	C26'—C25'—C24'	126 (3)
C22—N2—N1	105.2 (2)	C30'—C25'—C24'	114 (3)
C22—N2—H2	127.4	C25'—C26'—C27'	120.0
N1—N2—H2	127.4	C25'—C26'—Cl4'	120.7 (14)
C23—N3—C22	103.9 (2)	C27'—C26'—Cl4'	119.3 (14)
C46—N4—N5	102.1 (2)	C26'—C27'—C28'	120.0
C45—N5—N4	109.5 (2)	C26'—C27'—H27'	120.0
C45—N6—C46	102.8 (3)	C28'—C27'—H27'	120.0
C45—N6—H6	128.6	C29'—C28'—C27'	120.0
C46—N6—H6	128.6	C29'—C28'—H28'	120.0
C2—C1—Sn1	110.57 (19)	C27'—C28'—H28'	120.0
C2—C1—H1A	109.5	C28'—C29'—C30'	120.0
Sn1—C1—H1A	109.5	C28'—C29'—H29'	120.0
C2—C1—H1B	109.5	C30'—C29'—H29'	120.0
Sn1—C1—H1B	109.5	C29'—C30'—C25'	120.0
H1A—C1—H1B	108.1	C29'—C30'—H30'	120.0
C3—C2—C7	116.3 (3)	C25'—C30'—H30'	120.0
C3—C2—C1	122.7 (3)	C32—C31—Sn2	111.0 (2)
C7—C2—C1	121.0 (3)	C32—C31—H31A	109.4
C4—C3—C2	122.7 (3)	Sn2—C31—H31A	109.4
C4—C3—Cl1	118.2 (3)	C32—C31—H31B	109.4
C2—C3—Cl1	119.1 (2)	Sn2—C31—H31B	109.4
C5—C4—C3	119.4 (3)	H31A—C31—H31B	108.0
C5—C4—H4	120.3	C37—C32—C33	117.1 (4)
C3—C4—H4	120.3	C37—C32—C31	120.7 (4)
C6—C5—C4	119.5 (3)	C33—C32—C31	122.1 (3)
C6—C5—H5	120.3	C34—C33—C32	123.0 (4)
C4—C5—H5	120.3	C34—C33—Cl5	118.6 (3)
C5—C6—C7	120.5 (3)	C32—C33—Cl5	118.3 (3)
C5—C6—H6A	119.8	C33—C34—C35	118.0 (5)
C7—C6—H6A	119.8	C33—C34—H34	121.0
C6—C7—C2	121.6 (3)	C35—C34—H34	121.0
C6—C7—H7	119.2	C36—C35—C34	120.4 (4)
C2—C7—H7	119.2	C36—C35—H35	119.8
C9—C8—Sn1	112.0 (2)	C34—C35—H35	119.8
C9—C8—H8A	109.2	C35—C36—C37	120.5 (5)
Sn1—C8—H8A	109.2	C35—C36—H36	119.7
C9—C8—H8B	109.2	C37—C36—H36	119.7
Sn1—C8—H8B	109.2	C36—C37—C32	120.9 (5)
H8A—C8—H8B	107.9	C36—C37—H37	119.5
C10—C9—C14	116.0 (3)	C32—C37—H37	119.5
C10—C9—C8	122.8 (3)	C39—C38—Sn2	113.5 (4)
C14—C9—C8	121.2 (3)	C39—C38—H38A	108.9
C11—C10—C9	123.0 (3)	Sn2—C38—H38A	108.9
C11—C10—Cl2	118.2 (3)	C39—C38—H38B	108.9
C9—C10—Cl2	118.8 (2)	Sn2—C38—H38B	108.9
C10—C11—C12	119.2 (3)	H38A—C38—H38B	107.7
C10—C11—H11	120.4	C40—C39—C44	120.0
C12—C11—H11	120.4	C40—C39—C38	121.0 (5)
C13—C12—C11	119.5 (3)	C44—C39—C38	119.0 (5)
C13—C12—H12	120.2	C39—C40—C41	120.0
C11—C12—H12	120.2	C39—C40—Cl6	122.2 (2)
C12—C13—C14	120.1 (3)	C41—C40—Cl6	117.8 (2)
C12—C13—H13	119.9	C42—C41—C40	120.0
C14—C13—H13	119.9	C42—C41—H41	120.0
C13—C14—C9	122.0 (3)	C40—C41—H41	120.0
C13—C14—H14	119.0	C41—C42—C43	120.0
C9—C14—H14	119.0	C41—C42—H42	120.0
C16—C15—Sn1	109.2 (2)	C43—C42—H42	120.0
C16—C15—H15A	109.8	C44—C43—C42	120.0
Sn1—C15—H15A	109.8	C44—C43—H43	120.0
C16—C15—H15B	109.8	C42—C43—H43	120.0
Sn1—C15—H15B	109.8	C43—C44—C39	120.0
H15A—C15—H15B	108.3	C43—C44—H44	120.0
C21—C16—C17	116.1 (3)	C39—C44—H44	120.0
C21—C16—C15	120.7 (3)	C39'—C38'—Sn2	107.3 (8)
C17—C16—C15	123.1 (3)	C39'—C38'—H38C	110.3
C18—C17—C16	122.8 (3)	Sn2—C38'—H38C	110.3
C18—C17—Cl3	118.0 (2)	C39'—C38'—H38D	110.3
C16—C17—Cl3	119.2 (2)	Sn2—C38'—H38D	110.3
C19—C18—C17	118.9 (3)	H38C—C38'—H38D	108.5
C19—C18—H18	120.5	C40'—C39'—C44'	120.0
C17—C18—H18	120.5	C40'—C39'—C38'	118 (2)
C18—C19—C20	119.8 (3)	C44'—C39'—C38'	122 (2)
C18—C19—H19	120.1	C39'—C40'—C41'	120.0
C20—C19—H19	120.1	C39'—C40'—Cl6'	120.7 (7)
C21—C20—C19	120.2 (3)	C41'—C40'—Cl6'	119.2 (7)
C21—C20—H20	119.9	C42'—C41'—C40'	120.0
C19—C20—H20	119.9	C42'—C41'—H41'	120.0
C20—C21—C16	122.1 (3)	C40'—C41'—H41'	120.0
C20—C21—H21	118.9	C41'—C42'—C43'	120.0
C16—C21—H21	118.9	C41'—C42'—H42'	120.0
N2—C22—N3	112.1 (2)	C43'—C42'—H42'	120.0
N2—C22—S1	124.3 (2)	C44'—C43'—C42'	120.0
N3—C22—S1	123.6 (2)	C44'—C43'—H43'	120.0
N1—C23—N3	110.5 (3)	C42'—C43'—H43'	120.0
N1—C23—H23	124.7	C43'—C44'—C39'	120.0
N3—C23—H23	124.7	C43'—C44'—H44'	120.0
C25—C24—Sn2	113.4 (6)	C39'—C44'—H44'	120.0
C25—C24—H24A	108.9	N6—C45—N5	110.1 (3)
Sn2—C24—H24A	108.9	N6—C45—S2	126.8 (2)
C25—C24—H24B	108.9	N5—C45—S2	123.1 (2)
Sn2—C24—H24B	108.9	N4—C46—N6	115.4 (3)
H24A—C24—H24B	107.7	N4—C46—H46	122.3
C26—C25—C30	120.0	N6—C46—H46	122.3
			
C15—Sn1—S1—C22	117.76 (13)	C24—C25—C30—C29	176.0 (10)
C1—Sn1—S1—C22	−2.45 (13)	C31—Sn2—C24'—C25'	−132 (3)
C8—Sn1—S1—C22	−120.02 (14)	C38—Sn2—C24'—C25'	99 (4)
C31—Sn2—S2—C45	−58.74 (15)	C38'—Sn2—C24'—C25'	92 (4)
C38—Sn2—S2—C45	64.7 (2)	C24—Sn2—C24'—C25'	−72 (9)
C38'—Sn2—S2—C45	62.7 (6)	S2—Sn2—C24'—C25'	−15 (4)
C24'—Sn2—S2—C45	177.1 (19)	Sn2—C24'—C25'—C26'	84 (4)
C24—Sn2—S2—C45	−178.8 (6)	Sn2—C24'—C25'—C30'	−97 (3)
C23—N1—N2—C22	0.7 (3)	C30'—C25'—C26'—C27'	0.0
C46—N4—N5—C45	−0.5 (3)	C24'—C25'—C26'—C27'	178 (3)
C15—Sn1—C1—C2	98.9 (2)	C30'—C25'—C26'—Cl4'	−177 (2)
C8—Sn1—C1—C2	−25.5 (2)	C24'—C25'—C26'—Cl4'	1(2)
S1—Sn1—C1—C2	−141.5 (2)	C25'—C26'—C27'—C28'	0.0
Sn1—C1—C2—C3	89.6 (3)	Cl4'—C26'—C27'—C28'	177 (2)
Sn1—C1—C2—C7	−89.7 (3)	C26'—C27'—C28'—C29'	0.0
C7—C2—C3—C4	−1.2 (5)	C27'—C28'—C29'—C30'	0.0
C1—C2—C3—C4	179.5 (3)	C28'—C29'—C30'—C25'	0.0
C7—C2—C3—Cl1	178.8 (2)	C26'—C25'—C30'—C29'	0.0
C1—C2—C3—Cl1	−0.5 (4)	C24'—C25'—C30'—C29'	−178 (3)
C2—C3—C4—C5	1.8 (5)	C38—Sn2—C31—C32	158.0 (4)
Cl1—C3—C4—C5	−178.2 (3)	C38'—Sn2—C31—C32	165.8 (11)
C3—C4—C5—C6	−0.8 (5)	C24'—Sn2—C31—C32	31.9 (15)
C4—C5—C6—C7	−0.7 (5)	C24—Sn2—C31—C32	27.3 (5)
C5—C6—C7—C2	1.2 (5)	S2—Sn2—C31—C32	−82.0 (3)
C3—C2—C7—C6	−0.2 (4)	Sn2—C31—C32—C37	99.2 (4)
C1—C2—C7—C6	179.1 (3)	Sn2—C31—C32—C33	−77.8 (4)
C15—Sn1—C8—C9	−28.2 (3)	C37—C32—C33—C34	0.5 (6)
C1—Sn1—C8—C9	94.9 (2)	C31—C32—C33—C34	177.6 (4)
S1—Sn1—C8—C9	−148.4 (2)	C37—C32—C33—Cl5	−178.2 (3)
Sn1—C8—C9—C10	85.0 (3)	C31—C32—C33—Cl5	−1.1 (5)
Sn1—C8—C9—C14	−94.2 (3)	C32—C33—C34—C35	−0.3 (6)
C14—C9—C10—C11	−2.3 (5)	Cl5—C33—C34—C35	178.4 (3)
C8—C9—C10—C11	178.4 (3)	C33—C34—C35—C36	−0.6 (7)
C14—C9—C10—Cl2	178.1 (2)	C34—C35—C36—C37	1.4 (8)
C8—C9—C10—Cl2	−1.1 (4)	C35—C36—C37—C32	−1.2 (7)
C9—C10—C11—C12	2.2 (5)	C33—C32—C37—C36	0.3 (6)
Cl2—C10—C11—C12	−178.2 (2)	C31—C32—C37—C36	−176.9 (4)
C10—C11—C12—C13	−0.3 (5)	C31—Sn2—C38—C39	−150.9 (7)
C11—C12—C13—C14	−1.4 (5)	C38'—Sn2—C38—C39	106 (9)
C12—C13—C14—C9	1.3 (5)	C24'—Sn2—C38—C39	−19.3 (10)
C10—C9—C14—C13	0.6 (4)	C24—Sn2—C38—C39	−18.4 (9)
C8—C9—C14—C13	179.8 (3)	S2—Sn2—C38—C39	89.8 (7)
C1—Sn1—C15—C16	−27.4 (2)	Sn2—C38—C39—C40	78.8 (8)
C8—Sn1—C15—C16	95.2 (2)	Sn2—C38—C39—C44	−102.0 (6)
S1—Sn1—C15—C16	−146.19 (18)	C44—C39—C40—C41	0.0
Sn1—C15—C16—C21	−91.4 (3)	C38—C39—C40—C41	179.2 (5)
Sn1—C15—C16—C17	85.8 (3)	C44—C39—C40—Cl6	−178.9 (3)
C21—C16—C17—C18	−1.6 (5)	C38—C39—C40—Cl6	0.3 (5)
C15—C16—C17—C18	−178.9 (3)	C39—C40—C41—C42	0.0
C21—C16—C17—Cl3	178.5 (2)	Cl6—C40—C41—C42	178.9 (3)
C15—C16—C17—Cl3	1.1 (4)	C40—C41—C42—C43	0.0
C16—C17—C18—C19	0.8 (5)	C41—C42—C43—C44	0.0
Cl3—C17—C18—C19	−179.2 (3)	C42—C43—C44—C39	0.0
C17—C18—C19—C20	0.1 (5)	C40—C39—C44—C43	0.0
C18—C19—C20—C21	−0.3 (5)	C38—C39—C44—C43	−179.2 (5)
C19—C20—C21—C16	−0.5 (5)	C31—Sn2—C38'—C39'	−132 (2)
C17—C16—C21—C20	1.4 (5)	C38—Sn2—C38'—C39'	−52 (6)
C15—C16—C21—C20	178.8 (3)	C24'—Sn2—C38'—C39'	5(3)
N1—N2—C22—N3	−1.1 (3)	C24—Sn2—C38'—C39'	6(3)
N1—N2—C22—S1	179.1 (2)	S2—Sn2—C38'—C39'	112 (2)
C23—N3—C22—N2	1.1 (3)	Sn2—C38'—C39'—C40'	70 (2)
C23—N3—C22—S1	−179.1 (2)	Sn2—C38'—C39'—C44'	−107.1 (18)
Sn1—S1—C22—N2	−85.9 (3)	C44'—C39'—C40'—C41'	0.0
Sn1—S1—C22—N3	94.3 (2)	C38'—C39'—C40'—C41'	−177.1 (13)
N2—N1—C23—N3	0.0 (3)	C44'—C39'—C40'—Cl6'	178.4 (9)
C22—N3—C23—N1	−0.7 (3)	C38'—C39'—C40'—Cl6'	1.3 (12)
C31—Sn2—C24—C25	−132.8 (10)	Cl6'^i^—Cl6'—C40'—C39'	92.8 (12)
C38—Sn2—C24—C25	95.4 (12)	Cl6'^i^—Cl6'—C40'—C41'	−88.8 (13)
C38'—Sn2—C24—C25	88.8 (19)	C39'—C40'—C41'—C42'	0.0
C24'—Sn2—C24—C25	105 (13)	Cl6'—C40'—C41'—C42'	−178.4 (9)
S2—Sn2—C24—C25	−18.7 (13)	C40'—C41'—C42'—C43'	0.0
Sn2—C24—C25—C26	89.1 (12)	C41'—C42'—C43'—C44'	0.0
Sn2—C24—C25—C30	−87.0 (12)	C42'—C43'—C44'—C39'	0.0
C30—C25—C26—C27	0.0	C40'—C39'—C44'—C43'	0.0
C24—C25—C26—C27	−176.1 (10)	C38'—C39'—C44'—C43'	177.0 (13)
C30—C25—C26—Cl4	179.5 (8)	C46—N6—C45—N5	−0.8 (3)
C24—C25—C26—Cl4	3.3 (7)	C46—N6—C45—S2	178.7 (2)
C25—C26—C27—C28	0.0	N4—N5—C45—N6	0.9 (3)
Cl4—C26—C27—C28	−179.5 (8)	N4—N5—C45—S2	−178.7 (2)
C26—C27—C28—C29	0.0	Sn2—S2—C45—N6	−0.8 (3)
C27—C28—C29—C30	0.0	Sn2—S2—C45—N5	178.7 (2)
C28—C29—C30—C25	0.0	N5—N4—C46—N6	0.0 (3)
C26—C25—C30—C29	0.0	C45—N6—C46—N4	0.5 (4)

Symmetry codes: (i) −*x*+2, −*y*+2, −*z*+2.

### Hydrogen-bond geometry (Å, °)

**Table:**

*D*—H···*A*	*D*—H	H···*A*	*D*···*A*	*D*—H···*A*
N2—H2···N5	0.86	2.07	2.916 (4)	170
